# Obvious and Hidden Anxiety and the Related Factors in Operating Room Nurses Employed in General Hospital, Qazvin, Iran: A Cross-Sectional Study

**DOI:** 10.5539/gjhs.v5n6p202

**Published:** 2013-09-29

**Authors:** Hamid Kayalha, Zohreh Yazdi, Shahram Rastak, Mojtaba Dizaniha

**Affiliations:** 1 Dept of Anesthesiology, Qazvin University of Medical Sciences, Qazvin, Iran; 2 Metabolic Disease Research Center, Qazvin University of medical Sciences, Qazvin, Iran; 3 Dept of Paramedical Sciences, Qazvin University of Medical Sciences, Qazvin, Iran

**Keywords:** obvious anxiety, hidden anxiety, nursing, anesthesia technician, operating room technician

## Abstract

**Background:**

Health promotion and security of manpower in a society is one of the pillars to progress a society. Anxiety, is the most common psychological disorder in societies and occupations like nursing, anesthesia technicians and operation room technicians. The aim of this study was to investigate prevalence of anxiety, and its severity in Iranian nurses working in operation room. Also, we determined the most important associated factors with anxiety.

**Methods:**

In this cross sectional study, 152 nurses working in operating room participated. The tool to gather the data was a questionnaire, that included three parts; demographic information, obvious anxiety questions and hidden anxiety questions of Spielburger. Obtained data was analysed with SPSS 16 software.

**Results:**

The majority of participants were females (94.7%) with experience at work less than 10 years (84.9%). The average scores of participants in obvious and hidden anxiety were 41.9±39.4 (range 20 to 75) and 39.4±8.2 (range 20 to 70), respectively. Anxiety level was significantly higher in females than males (P=0.04). The most prevalent cause of anxiety, was contact with infected biological factors (23% of nurses). The less important cause was concern about retirement (42.8% of nurses).

**Conclusion:**

Our results suggest that, anxiety disorders is prevalent in Iranian nurses working in public city hospitals, which warrants immediate programs for intervention to improve working situations in work place.

## 1. Introduction

Anxiety is a known phenomenon in the community, which is shown at the different stages in people life with its positive and negative consequences. Anxiety, was explained for the first time in the fifteenth century as the physical pressure. Also, Banner in the psycho-physical program, used it as the cause of the disease in 17^th^ century (Hemati, 2005). Job is one of the most important causes of anxiety in individual’s lives (Rahimi, 2009; Mehrabi et al., 2005). Anxiety disorders appear to be pervasive, and increasingly prevalent in workplaces in recent years. The etiology of most anxiety disorders, is multifactorial so, in most cases a combination of several factors cause anxiety including; individual genetic, developmental, work and non-work causes. It seems that, both; work-related organizational-individual and, social risk factors interact. The interaction determines the onset, progression and course of anxiety disorders (Saki et al., 2002; Mattew et al., 2003).

According to this definition, there are many work conditions, tasks and demands, and/or related occupational stressors that, are associated with the onset of acute states of anxiety or manifestations of anxiety. Some of the occupational factors include; an overwhelming workload, pace of work, deadlines and a perceived lack of personal control (Timothy et al., 1999; Mesler et al., 2001; Murphy., 2002).

Individuals, employed as health care workers including; physicians and nurses, are exposed to anxiety for various reasons. Nurses are one of the largest cadres of work force in the health care system. Therefore, the quality of their performance affects the quality of patient care. Nurses working in the hospital, are known to work in a stressful environment. Some of the reasons that cause anxiety among nurses include; concerns about encountering with occupational hazards (e.g. infectious or injuries), an unequal position compare to other health care providers, working in irregular shifts, limited staffing resources, worries over potential medical accidents and the associated consequences, and psychological and physical exhaustion due to the intensive care of patients (Verna et al., 2000; Karasek, 1979).

Anxiety, has many detrimental effects on health and nursing performance. Nurses with high job anxiety, show decreased job satisfaction, increased absenteeism, and less job commitment (Khaksari, 2007; Diet et al., 2000). In a cross sectional study, conducted by Gao et al. (2012) in Chinese nurses, who worked in public city hospitals, self-reported anxiety was measured by Self-Rating Anxiety Scale. Their findings showed that, the prevalence of anxiety symptoms in nurses was 43.4%. The most factors associated with anxiety symptoms, were lower job rank and over commitment.

In Iran, anxiety is one of the most common mental disorders. A recent study, has been done in Iranian population aged 18 years and older showed that, the prevalence of anxiety disorders was about 5% (Ahmadvand et al., 2012). Also, studies done in other countries showed that, the prevalence and severity of anxiety in nurses is higher than general population. The prevalence of anxiety in different countries reported from “the lowest rate” (7%) in Japanese nurses to “the highest rate” (43%) in Iranian nurses (Gao et al., 2012). Despite this fact, very few researches have been done about Iranian nurses’ anxiety and its related factors.

Current research was done with the aim of assessment the prevalence of anxiety, and its related factors in nursing cadres (anesthesia, operation room technician and nursing) of Velayat hospital -a public city hospital in Qazvin.

## 2. Methods

A cross-sectional survey was carried out from February 2012 through March 2012 in Velayat hospital (a public city hospital) in Qazvin. Velayat hospital in Qazvin, is a referral teaching hospital with 250 beds.

Sampling was done by capitation and using the list of personnel that employment department of Qazvin Velayat hospital has handed to the researcher. All registered nurses, participated at the time of conducting the study. It is necessary to mention that, nursing cadre are a subset of nursing office (anesthesia, operation room technician and nursing). Ethical committee of Qazvin University of medical sciences, permitted doing this study. All the participants signed an informed consent form, provided by researchers. Also, Information was given to them about the voluntary participation and anonymity for the participants.

Data collection was done by using a questionnaire including 3 parts:

1- personal specifications (age, weight, height, body mass index, life mode, certificate, work acquaintance). 2- 20 questions about obvious anxiety and, 20 questions about hidden anxiety. 3- items that showed nursing views about most important factors, inducing; anxiety in the workplace. They scored related factors with anxiety on a four-point Likert scale, from “most important” to “least important”. These factors include; exposure to infectious biologic factors, conflict with supervisor, conflict with co-workers, job insecurity, high job demands, and retirement.

Data collection about hidden and obvious anxiety, was done by the standard Spielburger questionnaire, which is known as Spielburger State-Trait Anxiety Inventory (STAI) (Spielberger, 1979). In 1993, a study was done, in order to standardization the Spielburger’s test by Mahram. He studied the final rate in both normal and criteria groups, separately. Final rate in the index of hidden and obvious anxiety on the basis of Cranbach alpha, is 0/9084, and 0/9025 respectively (Mahram, 1993). The time needed to complete the questionnaire was about 30 minutes. Content validity of questionnaires, determined by a panel including; three psychiatrics and two psychiatric nurse professionals. Also, reproducibility assessed through Cronbach alpha coefficient.

The data derived from the study were processed by statistic software SPSS 16. Descriptive data expressed as, mean ± standard deviation. Differences between means, were compared using student t-test. Chi-square test was used to estimate differences in categorical data. The correlation between quantitative parameters computed by Individual correlation. ANOVA One-way, used to compare means among more than two groups. Reliability of the questionnaire in Iranian population was calculated by” Cronbach Alpha coefficient”, and two-week test retest correlation. P-value less than 0.05 was considered significant, statistically.

## 3. Results

152 questionnaires among 195 ones under gone analysis. Forty three questionnaires were having imperfect, and unreadable response -so response rate was 77.9%. Table 1, shows the characteristics of the participants. As the table shows, the majority of participants were female (94.7%) with less than 10 years (84.9%) of work experience.

**Table 1:** Demographic characteristics of the study participants (n=152)

Percentage	Number	Demographic
**Sex**		
Male	8	5.3%
Female	144	94.7%
**Age**		
≤ 30	110	72.4%
>30	42	27.6%
BMI*		
Less Than Normal	23	15.2%
Normal	97	63.8%
Overweight and Obese	32	21%
**Experience at work**		
Less Than 10 Years	129	84.9%
More than 10 Years	23	15.1%
**Income**		
Less Than 1 Million Toman**	114	75%
More Than 1 Million Toman	7	4.6%
**Marriage**		
Single	53	34.9%
Married	99	65.1%
**Level of education**		
Less than 12 years	15	9.9%
More than 12 years	137	90.1%
**Type of Certificate**		
Technicians (Anesthesia and operation room)	22	14.5%
Nurses	130	85.5%
**Professional cadre**		
Technical nurses	131	86.2%
Head Nurses and superior Nurses***	21	13.8%
**Shifts**		
Rotating shift	84	55.3%
Fixed shift	32	21.1%

* Less than normal: BMI<18.5; Normal: 18.5-24.9; Overweight and obese: BMI≥25

** This amount is equal with 500$ within the time questions were asked

*** nurses with managerial role

The average scores of participants in obvious and hidden anxiety, were 41.9±39.4 (range between 20 and 75) and 39.4±8.2 (range between 20 to 70), respectively. Table 2, shows the distribution of nursing cadre according to different severities of anxiety. As the table shows, about half of the participants are located above intermediate level of anxiety at both; the obvious and hidden category.

**Table 2:** Distribution of different levels of anxiety in participants in study (n=152)

Genera Of Anxiety		Obvious Anxiety	Hidden Anxiety	Total
**Low**	Number	27	26	53
	Percentage	17.8%	17.1%	17.4%
**Under Intermediate**	Number	55	68	123
	Percentage	36.2%	44.7%	40.4%
**Upper Intermediate**	Number	54	48	101
	Percentage	35.5%	31.6%	33.2%
**Rather Severe**	Number	13	9	21
	Percentage	8.6%	5.9%	6.9%
**Severe**	Number	3	1	4
	Percentage	1.9%	0.7%	1.3%

The most prevalent cause of anxiety was contact with infected biological factors (23% of nurses). The less important cause was concern about retirement (42.8% of nurses).

Table 3 shows association between socio-demographic variables with nurse’s anxiety. As the table show, there is a significant difference in anxiety between male and female participants. Anxiety level was significantly higher in female, than male (P=0.04).

**Table 3:** Compare the level of obvious and hidden anxiety, among nursing cadre with different demographic, and work condition

P-value	Obvious anxiety	P-value	Hidden anxiety	Variables
**Sexuality**				
Male	38.2±9.4	0.3	34±4.8	0.04
Female	42.1±10.5		39.7±8.2	
**Marriage**				
Single	42.2±11.2	0.6	39.2±8.6	0.72
Married	41.4±8.8		39.7±7.4	
**Type of Certificate**				
Technicians (Anesthesia and operation room)	40.8±8.6	0.6	38.8±6.9	0.7
Nurses	42.1±10.7		39.6±8.4	
**Situation in Organization**				
Technical Personnel	43.6±6.3	0.2	39.9±7.8	0.1
Head Nurses and superior nurses	39.7±5.9		37.2±5.8	
**Shifts**				
Rotating shift	41.5±10.6	0.2	39.5±8.4	0.9
Fixed morning shift	43.8±9.7		30.4±7.5	

In examining the correlation between obvious anxiety with age it was shown that, with increasing age, obvious and hidden anxiety decrease significantly in nurses (ρ=-0.2, P=0.03, and ρ=-0.8, P=0.02, respectively). Also, there was a significant reverse correlation between BMI and obvious anxiety. Thus, with increasing BMI, obvious anxiety decreases in nursing cadre (ρ=-0.6, P=0.04). There were no correlation between experience at work, income and level of education with obvious and hidden anxiety.

## 4. Discussion

Results from this study showed that, about half of the participants experienced above intermediate level of anxiety, at both; obvious and hidden category. Working as a health care worker, is considered as one of the most dangerous jobs, different types of hazardous exposure such as; needle stick injuries, physical and chemical exposure, ergonomic factors and psychological factors such as; irregular work and temporary work.

In a study by Samadpour and Kianmehr in 2006, they concluded that, patient’s pain and death is the most important factor in prevailing stress. Also, their study showed that, nurses who were working at internal disease and surgery wards experienced maximum level of anxiety compared with another wards of hospital. Whereas in our study, the most important reason of anxiety was exposure with biologic factors at the workplace. Rahimi and colleagues in 2004 conducted a study in order to know the range of anxiety and related factors in nurses from Tehran hospitals. From all those samples 44.1%, 54.1 and 1.8% had experiences high, moderate and low level of anxiety, respectively.

Etemadi et al. in 2004 evaluated relationship between occupation-related stress with nurses’ job satisfaction at CCU and heart diseases wards in Tehran University of medical science. They concluded that stress level of the nurses working for less than one year and young nurses were significantly higher than other nurses. Another study in Hamedan by Khodaveisi and colleagues in 2005, the maximum stress was seen in young nurses aged between 20-24 years old. Also, Married nurses had high stress compared with the single ones. In this study, there was a strong relationship between anxiety and age, so that, with increasing age, obvious and hidden anxiety decrease, significantly.

Another study conducted by Kolakari and colleagues in 2002, assessed stress level among operation room personnel in Gorgan’s hospitals. They concluded that, the majority of participants suffered from low level of stress (54.4%). Also, the most important factors related to stress was “unpleasant smelling” in operating room (76% of participants), and the least important factors was interrelationship with their colleagues (29.7% of paticipants). While, in this study, the most important cause for anxiety was concern about encountering with infected biologic factors. All health care providers in hospitals, especially nurses in Iran, are dealing with health-care issues related to the spread of serious infectious diseases, including: hepatitis B and C, severe acute respiratory syndrome, and HIV. In recent years, many efforts has been performed to reduce the risk of disease transmission to nurses at work place. However, the risks of being infected with these diseases are considered as one of the most important occupational disease yet (Costigliola et al., 2012).

Another factor related to anxiety in our study, was the age of participants. Nursing cadre with older age had suffered lower level of anxiety compared to younger ones. Results from other studies are contradictory in this case. A cross-sectional study conducted by Boya et al. (2008) didn’t find any relationship between age and anxiety in nurses. In our study, there was no significant relationship between level of education and anxiety. Navidian and colleagues in 2005 had shown that, with increasing level of education, anxiety increase significantly in nursing cadre (P=0.02). More study is needed to explain relationship between age and level of education with anxiety.

According to results from our study, it is recommended to pay more attention to different risk factors of anxiety in nursing cadre, including infected biologic factors. Employers have a responsibility to minimize risk of hazardous exposure in the work setting.

## 5. Conclusion

According to our results, we demonstrated high prevalence of anxiety in nurses employed in the operating room. Also, anxiety level was significantly higher in younger female nurses, and the most important cause of anxiety was contact with infected biologic factors for nurses. Moderating proper strategies to reduce anxiety, is necessary for hospitals. These strategies include; education, providing adequate facilities for consultation, promotion of healthy lifestyle practices, and improving social environments of work places.
